# ﻿Karyotypic analysis and isolation of four DNA markers of the scleractinian coral *Favitespentagona* (Esper, 1795) (Scleractinia, Anthozoa, Cnidaria)

**DOI:** 10.3897/compcytogen.v16.i1.79953

**Published:** 2022-04-05

**Authors:** Rei Kawakami, Takahiro Taguchi, Joshua Vacarizas, Masumi Ito, Takuma Mezaki, Akira Tominaga, Satoshi Kubota

**Affiliations:** 1 Agriculture and Marine Science Program, Graduate School of Integrated Arts and Sciences, Kochi University, Kohasu, Oko-Cho, Nankoku, Kochi 783-8505, Japan; 2 Department of Nutrition, Faculty of Health Science, Kochi Gakuen University, 292-26 Asahitenjin-Cho, Kochi 780-0955, Japan; 3 Kuroshio Science Program, Graduate School of Integrated Arts and Sciences, Kochi University, Kohasu, Oko-Cho, Nankoku, Kochi 783-8505, Japan; 4 Faculty of Agriculture and Marine Science, Kochi University, 200 Otsu, Monobe, Kochi 783-8502, Japan; 5 Kuroshio Biological Research Foundation, Otsuki, Hata County, Kochi 788-0333, Japan; 6 Kuroshio Science Unit, Multidisciplinary Science Cluster, Kochi University, Kohasu, Oko-Cho, Nankoku, Kochi 783-8505, Japan; 7 Graduate School of Fisheries Sciences, Hokkaido University, 3-1-1 Minato-Cho, Hakodate, Hokkaido, 041-8611, Japan

**Keywords:** chromosome, FISH, histone, HSR, karyotype, rRNA, scleractinian coral

## Abstract

We performed conventional and molecular cytogenetic studies on the *Favitespentagona* Esper, 1795, a scleractinian coral mostly found along the west coast of Japan. Karyotype analysis of *F.pentagona* by G-banding revealed a karyogram containing a homogenously staining region (HSR) on chromosome 10 in more than 50% of the examined metaphase spreads. This HSR consisted of sequences from 18S ribosomal RNA (rRNA) genes, as demonstrated by fluorescence in situ hybridization (FISH) and DNA sequencing. We highlighted the development of four chromosomal FISH markers from repetitive genes such as U2 small nuclear RNA linked to 5S rRNA sequence (U2 snRNA-5S), 18S rRNA, histone H3, and uncharacterized gene FP-9X. The chromosomal locations of the U2 snRNA-5S and 18S RNA were on the terminal end of long arm of chromosomes 2 and 10, respectively, while the histone H3 and the uncharacterized gene were located near the centromeres of chromosomes 1 and 9, respectively. These FISH markers will improve the karyotyping of *F.pentagona* from mitotic preparations which helps in widening our understanding of coral genetic structure and chromosome organization. In addition, these improvements in karyotyping will provide the basis in constructing of chromosome-level genome assembly for *F.pentagona*.

## ﻿Introduction

Cytogenetic information from karyotypic analysis gives us a deeper understanding the way genetic material is packaged inside a nucleus of a cell and how certain genetic diseases are associated with chromosome defects and aberrations (Testa 1990; Super 1991). Cytogenetic information is also valuable in understanding the genome structure and organization of an individual which may vary greatly within and across species (Sullivan 2020). These variations in chromosome characteristics will give insight into their evolutionary process as they reflect genome shuffling, translocation, and chromosomal duplication/deletion (Guo et al. 2018). This information might be valuable for stony corals, in which taxonomic classification poses a great challenge as the integration of its morphological and molecular characteristics often reveal conflicting results (Fukami et al. 2004). This difficulty might be caused, in part, by hybridization of closely related species and by morphological plasticity. Confusion about the stony coral taxonomy necessitates the search for new coral characters such as cytogenetic information to understand coral systematics and evolution. However, the cytogenetic information of stony corals, such as karyotypes and gene maps, is limited. To date, molecular cytogenetic information on stony corals has only been reported for five species from three different families (2 species from Acroporidae, 2 species from Merulinidae, and 1 species from Lobophyliidae). These are *Acroporasolitaryensis* Veron et Wallace,, 1984 and *Acroporapruinosa* Brook, 1893 (Acroporidae), *Coelastreaaspera* Verrill, 1866 and *Platygyracontorta* Veron, 1990 (Merulinidae), and *Echinophylliaaspera* Ellis et Solander, 1786 (Lobophylliidae) (Taguchi et al. 2013, 2014, 2016, 2017, 2020; Vacarizas et al. 2021). In those studies, new cytogenetic evidence was presented, including information regarding chromosome numbers, rRNA gene loci, the presence of a homogenously staining regions (HSR), and some repeated sequences shared with human satellite DNA.

In this study, we reported the detailed molecular cytogenetic analysis of stony coral *Favitespentagona* Esper, 1758, from family Merulinidae. *F.pentagona* is commonly observed along the west coast of Japan (Veron, 2000). Colonies of *F.pentagona* range from massive, encrusting to columnar forms. The valleys with colonies are usually long and relatively straight at colony margins, becoming increasingly short, sinuous, and contorted towards the colony center; septa have thin walls and are highly irregular (Veron, 2000).

Cytogenetic analysis of *F.pentagona* was conducted using conventional and molecular cytogenetic techniques, such as fluorescence in situ hybridization (FISH). We identified a homogenously staining region (HSR) on *F.pentagona* chromosome 10 using G-banding and 4’,6-diamidino-2-phenylindole (DAPI) staining, followed by karyotyping. Furthermore, the chromosomal locations of four tandemly repetitive genes were identified for *F.pentagona*. The development of four FISH markers was described, and the FISH signals of each gene were characterized showing the effectivity of these markers to identify specific chromosomes from mitotic cells.

## ﻿Materials and methods

### ﻿Coral collection

**Figure 1.**
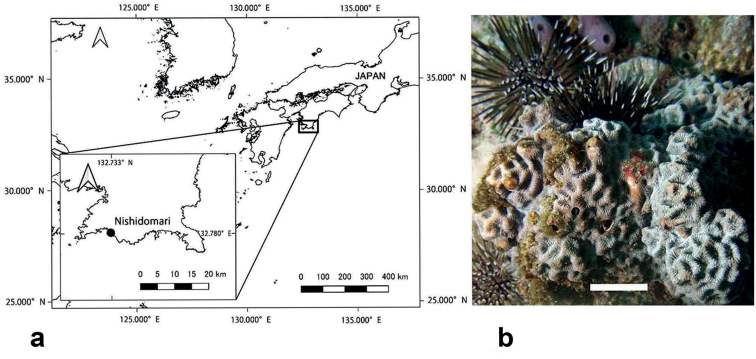
Map of the sampling site of *Favitespentagona* in Shikoku Island. Nishidomari, Otsuki-cho, Hata county, Kochi 788-0333, Japan (32°,46'44"N, 132°43'57"E) (**a**). Inset: Enlarged image of the collection area. Appearance of *Favitespentagona* in the sea (**b**). Scale bar: 2 cm.

*F.pentagona* gametes were collected from spawning colonies at Nishidomari (32°46'N, 132°43'E), Kochi Prefecture, Japan (Fig. 1). The release of gamete bundles was observed between 8:00 pm and 9:30 pm on July 24, 2019. Coral bundles were collected using plastic cups placed over the colonies during spawning. After collection, eggs and sperm from the spawned bundles were separated. The separated gametes were then transferred to a new container to allow fertilization. Successful cell divisions were observed under the light microscope. Embryos were then rinsed in 0.2 µm filtered seawater (ADVANTEC cartridge filter; Advantec Toyo Corp., Tokyo, Japan) to remove external contaminants.

### ﻿Chromosome preparations and G-banding

Coral chromosome preparations were conducted according to the method described by Taguchi et al. (2013). About 10–14 hours after artificial fertilization, embryos were treated in filtered seawater supplemented with 0.01% (v/v) colchicine (Sigma, St. Louis, MO, USA) for 1 h, followed by treatment with hypotonic solution (0.5 × sea water; diluted in distilled water) to spread the chromosomes. Embryos were fixed using a freshly prepared fixative containing absolute methanol and glacial acetic acid (3:1). Fixed embryos were soaked in diethyl ether overnight to remove intracellular lipids and incubated again in the fixative. Approximately 20 to 50 embryos were isolated using a fine needle to tear the embryos apart into their constituent cells. Suspensions containing embryo cells were transferred into a 1.5 ml tube filled with the fixative. The tube was centrifuged at 2000 *g* for 2 min and the pellet was re-suspended in 0.5 ml of fresh fixative. A drop containing separated cells was placed on a clean slide and then air- or flame-dried to spread the chromosomes. For G-banding, slides were treated with 0.025% trypsin solution at room temperature (approximately 25 °C) for 1 min (Seabright, 1973) and then stained with 5% Giemsa.

### ﻿DNA extraction

Genomic DNA was extracted from *F.pentagona* sperm (approximately 0.1 ml) using a Wizard Genomic DNA Purification Kit (Promega Corporation, Madison, WI, USA) according to the manufacturer’s instructions.

### ﻿PCR, DNA cloning and FISH probe preparation

Target genes (18S rRNA, U2 snRNA-5S, histone H3, and uncharacterized gene FP-9X) were amplified by PCR using the Emerald PCR master mix (Takara, Japan). The primer sets used are shown in Table 1. PCR was performed in a thermal cycler (WK-0518, Wako, Osaka, Japan) under the following conditions: initial denaturation for 2 min at 98 °C, followed by 30 cycles of 98 °C for 10 s, 55 °C for 30 s, and 72 °C for 1 min. PCR products were ligated into the pGEM-T Easy Vector (Promega, Madison, USA) and 30 ng of the ligation products were used to transform competent cells (JM109, pGMT-T Easy-Vector Systems, Promega). Transformed cells were plated onto Luria broth (LB) plates containing 100 μg/ml ampicillin, 40 μg/ml 5-Bromo-4-Chloro-3-Indolyl-β-D-Galactoside (X-Gal) and 0.05 mmol/L isopropyl-β-D-thiogalactopyranoside (IPTG). Isolated colonies were screened by FISH. Probes using FISH screening were prepared by random prime labeling with digoxigenin-12-dUTP (DIG-dUTP) or cyanine-3-dUTP (Cy3-dUTP) in accordance with the kit protocol (Invitrogen, Tokyo, Japan). Then, FISH-positive clones were later transferred into 15 ml test tubes containing 1.5 ml of LB/ampicillin medium and grown at 37 °C overnight. Plasmids from the resulting clones were extracted according to the manufacturer’s protocol using a Mini Plus Plasmid DNA Extraction System (Viogene, NACALAI TESQUE, INC., Kyoto, Japan).

**Table 1.** PCR primer sets used in this study.

Primer set	Genes	Sequence (5’-3’)	Reference	Species
1	18S rRNA	F-GGTTGATCCTGCCAGTAGTCATATGCTTG	Rowan and Powers (1992)	Zooxanthella
		R-GATCCTTCCGCAGGTTCACCTACGGAAACC		
2	histone H3	F-ATGGCTCGTACCAAGCAGACVGC	Huang et al., (2011)	Stony coral
		R-ATATCCTTRGGCATRATRGTGAC		
3	U2 snRNA-5S	F-CTTCCGTGATCGGACGAGAA	Stover and Steele (2001)	Hydra
		R-TATAATATTGGAACAGAATT		
4	Uncharacterized gene FP-9X	F-CTTCCGTGATCGGACGAGAA	Stover and Steele (2001)	Hydra
		R-CCAATTTTGTAGACATCTTGAAG	Kawaida et al., (2010)	Hydra

### ﻿DNA sequencing and homology search

DNA inserts from plasmids were sequenced with the M13 forward and reverse primers using the BigDye Terminator v3.1 Cycle Sequencing Kit (GE Healthcare, Japan) and ABI PRISM 3130 Genetic Analyzer (Thermo Fisher Scientific, Tokyo, Japan). DNA sequences were aligned, and homology searches were performed using Gapped BLAST and PSI-BLAST: a new generation of protein database search programs to search the GenBank database (http://www.ddbj.nig.ac.jp).

### ﻿FISH analysis

FISH analysis was performed as previously reported (Taguchi et al. 1993), with slight modifications. Metaphase preparations were denatured in 70% formamide/2x Saline-sodium citrate (SSC) at 70 °C for 2 min; 0.8 μl of the prepared probe was mixed with 10 μl of hybridization solution (H7782, Sigma, Japan) and denatured at 82 °C for 10 min. Hybridization was performed at 37 °C in a CO_2_ incubator for 12–15 h. After hybridization, samples were washed twice in 2x SSC and 1x Phosphate buffered detergent (PBD; 0.05% Tween20/4xSSC). Chromosomes were counterstained with DAPI.

### ﻿Image acquisition and processing

FISH slides were examined under a fluorescence microscope (Olympus BX-50, Tokyo, Japan) equipped with a cooled charge-coupled device. Images of suitable metaphase spreads were acquired using an Olympus DP70 workstation and the FISH analysis software. The mirror units used for each fluorescence light (FITC, Cy-3, and DAPI) were U-NIBA, U-MWU, and U-MWIB (Olympus), respectively.

## ﻿Results

### ﻿Diploid karyotypes in *F.pentagona*

**Figure 2.**
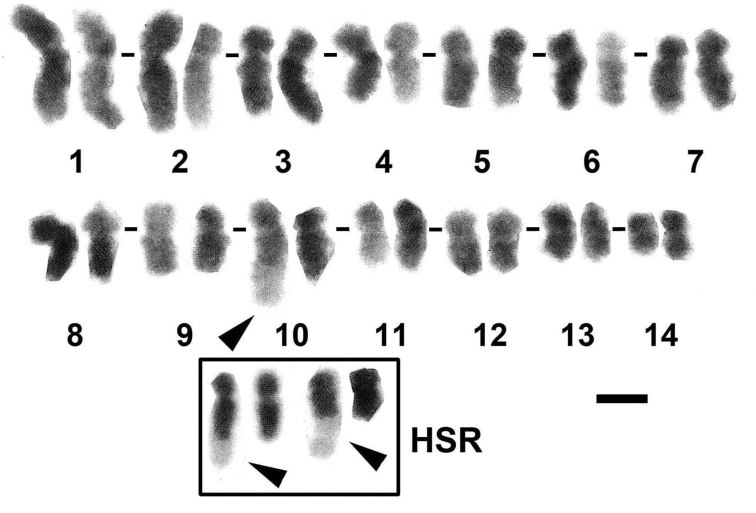
G-banded karyogram of *Favitespentagona* (2n = 28). The arrow indicates an HSR. Inset: two chromosome pairs with HSRs from two other metaphase spreads stained with DAPI (inverted images). One of the homologues has an HSR in each pair (arrowheads). Scale bar: 2 μm.

Chromosomes in metaphase cells were karyotyped by conventional trypsin G-banding, and an HSR in terminal end of one of the chromosomes was observed in approximately 50% of the observed metaphase spreads (Fig. 2). HSRs were shown in G-banded karyograms as long and lightly stained region of the chromosomes. The HSRs were also revealed using the inverted images of DAPI fluorescent staining showing the similar characteristics (Fig. 2, inset). Chromosomes were arranged in decreasing order of chromosome length from 1 to 14, which revealed that the chromosome with HSR is chromosome 10 (Fig. 2). The length and arm ratio of chromosomes were measured using eight metaphase spreads, as summarized in Table 2. The modal number of chromosomes per metaphase spread, which determined from 100 examined *F.pentagona* cells, was 28 (2n = 28). The percentage of metaphase spreads with an HSR from all cells was greater than 50% (43/80). Based on the arm ratio (Levan et al. 1964), this karyogram consisted of six submetacentric (1, 2, 3, 4, 5 and 7) and eight metacentric (6, 8, 9, 10, 11, 12, 13, and 14) chromosomes (Table 2).

**Table 2.** Relative lengths and centromere indices of the 14 chromosome pairs, shown as means and standard deviations obtained from the eight metaphase spreads.

Chromosome number	Short arm (μm)	Long arm (μm)	Total length (μm)	Arm ratio	Overall length ratio	Chromosome type*
1	2.21±0.75	3.94±0.95	6.15±1.5	1.88±0.57	0.94±0.08	sm
2	1.52±0.48	3.3±0.82	4.82±1.12	2.3±0.71	0.74±0.06	sm
3	1.55±0.52	2.91±0.83	4.46±1.08	1.99±0.62	0.68±0.04	sm
4	1.48±0.53	2.74±0.62	4.22±0.98	1.98±0.61	0.65±0.04	sm
5	1.41±0.35	2.63±0.74	4.04±0.95	1.92±0.48	0.62±0.03	sm
6	1.49±0.26	2.36±0.74	3.85±0.9	1.6±0.38	0.59±0.03	m
7	1.39±0.26	2.34±0.62	3.74±0.8	1.7±0.36	0.57±0.04	sm
8	1.47±0.38	2.15±0.43	3.62±0.79	1.49±0.17	0.56±0.03	m
9	1.32±0.28	2.14±0.51	3.46±0.73	1.64±0.32	0.53±0.04	m
10	1.26±0.23	2.05±0.49	3.31±0.67	1.63±0.26	0.51±0.04	m
11	1.31±0.3	1.9±0.41	3.21±0.63	1.49±0.33	0.5±0.04	m
12	1.2±0.34	1.85±0.4	3.05±0.66	1.6±0.33	0.47±0.04	m
13	1.12±0.23	1.53±0.35	2.65±0.49	1.41±0.38	0.41±0.04	m
14	0.94±0.23	1.25±0.27	2.2±0.45	1.37±0.31	0.34±0.05	m

*Types were categorized according to the reference of Levan et al. (1964). m: metacentric, sm: submetacentric. The part of an HSR on chromosome 10 was excluded.

### ﻿Physical mapping of four FISH markers

**Figure 3.**
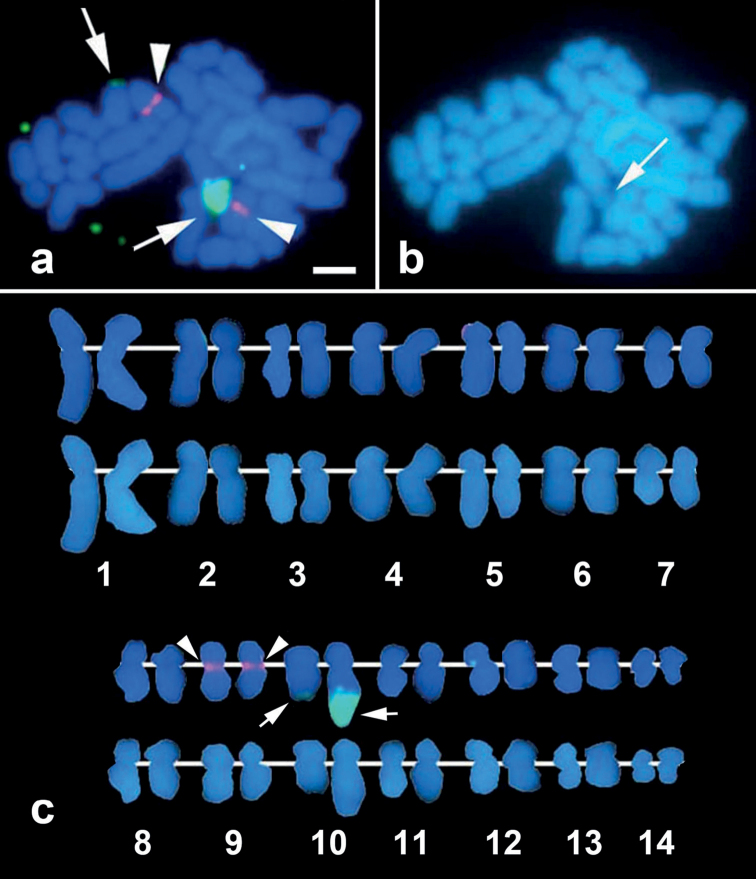
FISH image showing hybridization signals of uncharacterized gene FP-9X probe (red; arrowheads) and 18S rRNA gene probe (green; arrows) (**a**). Chromosomes were karyotyped according to size and centromere positions showing uncharacterized gene FP-X9 and 18S rRNA gene probe hybridization on chromosome 9 and chromosome 10, respectively (**c**, above alignment). DAPI-only channel revealing the HSR region pointed by the arrow (**b**) and its karyogram (**c**, below alignment). Scale bar: 2 μm.

**Figure 4.**
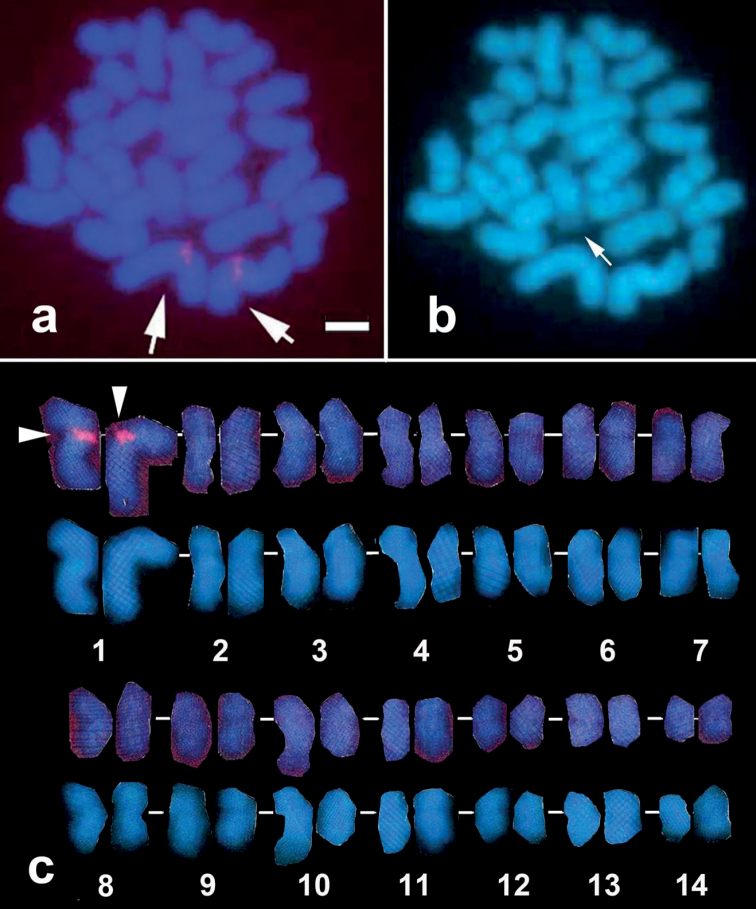
FISH image showing hybridization signal of histone H3 gene probe (red; arrowheads) (**a**). Chromosomes were karyotyped according to size and centromere positions showing the hybridization signal on chromosome 1 (**c**, above alignment). DAPI-only channel revealing the HSR region pointed by the arrow (**b**) and its karyogram (**c**, below alignment). Scale bar: 2 μm.

**Figure 5.**
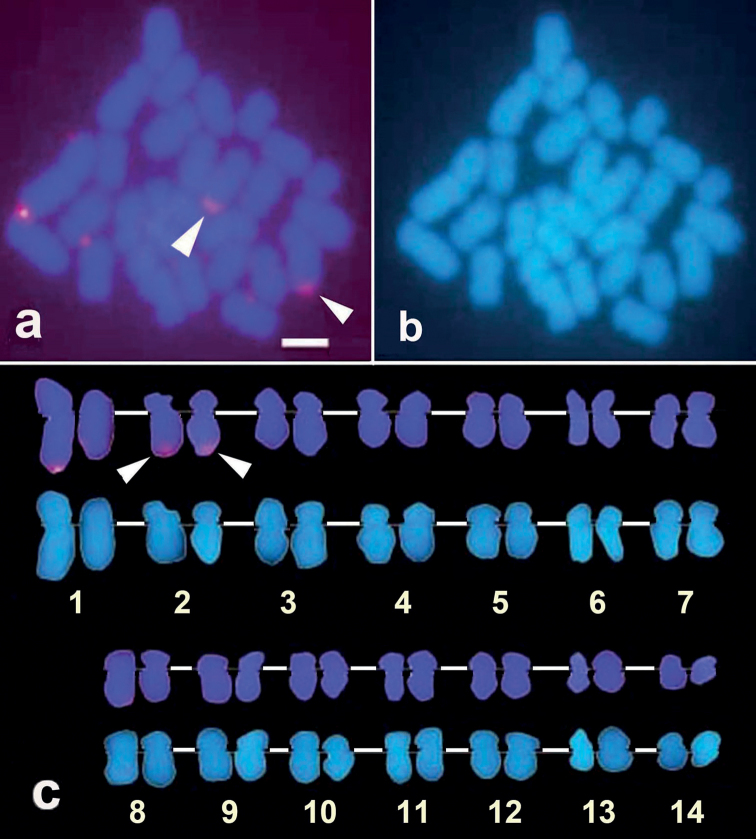
FISH image showing hybridization signal of U2 snRNA-5S gene probe (red; arrowheads) (**a**). Chromosomes were karyotyped according to size and centromere positions showing hybridization signal on chromosome 2 (**c**, above alignment). Few background signals were seen; one background signal was on long arm telomere of chromosome 1 (**a** and **c**). DAPI-only channel without HSR region (**b**) and its karyotype (**c**, below alignment). Scale bar: 2 μm.

FISH signals for the 18S rRNA gene locus were identified at the terminal ends of the long arm of chromosome 10 (Fig. 3a, c). The HSR, previously observed on chromosome 10 by G-banding, is hybridized by 18S rRNA gene probe, exhibiting a broad and intense hybridization signal (Fig. 3a and c, green signals indicated by arrows). The HSR was also recognized as a pale part of chromosome 10 in DAPI staining metaphase (Fig. 3b, arrow). FISH probe from the uncharacterized gene FP-9X was mapped on the centromeres of chromosome 9 (Fig. 3a, c, red signals indicated by arrowheads,). The histone H3 gene, on the other hand, was mapped near the centromere of the long arm of chromosome 1 (Fig. 4a, c), whereas the U2 snRNA-5S gene locus was mapped on the terminal ends of the long arm of chromosome 2 (Fig. 5a, c).

### ﻿Cloning and Sequence analysis of FISH probes

**Figure 6.**
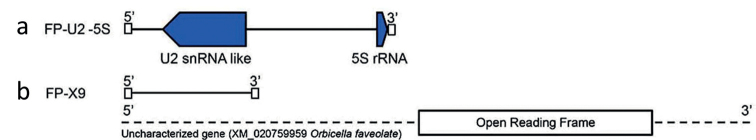
Schematic diagram of FISH probe sequences of FP-U2-5S which contains the U2 spliceosomal snRNA-like region and partial sequence of 5S rRNA gene sequence (**a**). FP-X9 is similar to the 5'-untranslated region of uncharacterized gene of a Merulinidae coral (*Orbicellafaveolata* (Ellis et Solander, 1786)) (**b**). Rectangles in both ends of bars show the primer positions and relative lengths.

**Figure 7.**
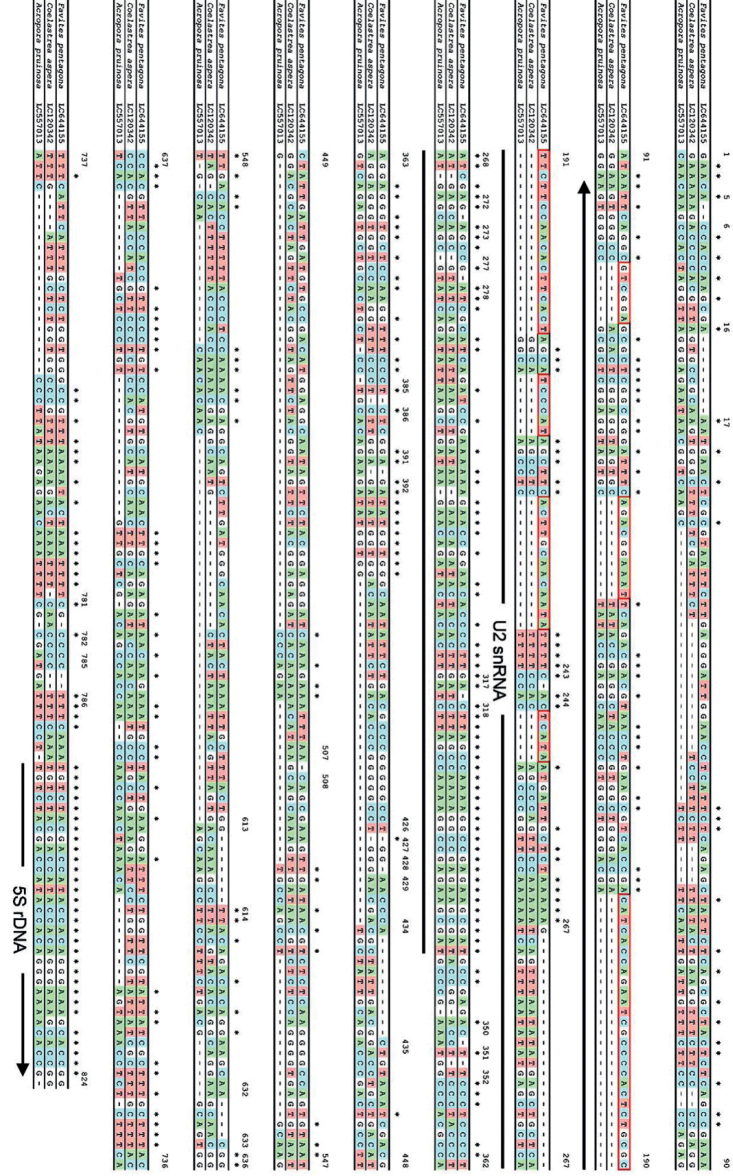
Comparison of FP-U2-5S sequence to *Coelastreaaspera* (Acession No. LC120342) and *Acroporapruinosa* (Acession No. LC557013) sequences. 5S rDNA sequences are highly conserved (793–823). There are 6 inserts in FP-U2-5S sequence (red rectangles: 102–107, 125–134, 165–208, 213–218, 225–238 and 246–250).

Cloning and sequencing were performed for the amplicons from which FISH probes were prepared. Positive clones were designated as FP-18S for 18S rRNA gene, FP-H3 for histone H3 gene, FP-U2-5S for U2 snRNA-5S, and FP-X9 for uncharacterized gene FP-X9. Sequence analysis of FP-18S clone (1,732 bp) with the GenBank database revealed a difference of a single nucleotide from the partial sequence of *F.pentagona* 18S rRNA gene (Accession No. LC644154). The FP-H3 clone (329 bp) completely matched with the partial sequence of the *F.pentagona* histone H3 gene (Accession No. LC644156). The FP-U2-5S (824 bp) sequence contained U2 spliceosomal small nuclear RNA (snRNA) gene sequence and region of 5S rRNA gene (Accession No. LC644155) (Figs 6A, 7). Lastly, the FP-X9 (357 bp) (Accession No. LC644157) showed homology to the 5'-untranslated region (UTR) of the uncharacterized mRNA of *Orbicellafaveolata* (Accession No. XM020759959) (Fig. 6B).

## ﻿Discussion

To solve the difficulties in taxonomically classifying stony corals (Fukami et al. 2004) and promote research on coral genetics, we have accumulated molecular cytogenetic data on several coral species. At this point, we have published six molecular cytogenetic reports with both conventional and molecular cytogenetic analyses of scleractinian corals, such as *Acroporasolitaryensis*, *Acroporapruinosa*, *Echinophylliaaspera*, *Coelastreaaspera*, and *Platygyracontorta* (Taguchi et al. 2013, 2014, 2016, 2017, 2020; Vacarizas et al. 2021), which are commonly found on the western coast of Japan (Wallace, 1999). The present study focuses on *F.pentagona*, belonging to the genus *Favites* and the family Merulinidae.

We carried out conventional G-bandings to establish the karyotype of *F.pentagona* embryos. In general, obtaining high-quality G-banding in invertebrate chromosomes is difficult because of the relatively small chromosome size and the weak effect of trypsin on G-banding. Karyotyping of *F.pentagona* revealed the three chromosome groups that cannot be precisely identified because of their similar lengths. Furthermore, differences in chromosome condensation depending on the stage of the cell cycle at which cells were fixed sometimes made it difficult to measure the precise lengths of chromosomes. To develop a coral chromosome study, it is necessary to identify each chromosome precisely using a painting probe (Gokhman et al. 2019). Nonetheless, G-banded chromosomes revealed the presence of HSRs. HSRs are created by the amplification and accumulation of certain DNA region within chromosomes. The amplification can be detected by conventional G-banding and FISH using locus-specific probes, which show intense hybridization signals on a single chromosome, as opposed to two copies with normal-homologous chromosomes (Biedler and Spencer 1976). In this study, we demonstrated that the HSR in the *F.pentagona* chromosomes is composed mainly of sequences from 18S rRNA gene. Generally, genetic amplification at the chromosomal level is manifested in the form of HSRs in tumor cells (Takaoka et al. 2012). We arranged each chromosome in decreasing order of their lengths and located the long chromosome 10 with an HSR. In our previous studies (Taguchi et al. 2013, 2016, 2017), using G-banding and FISH analyses, we revealed the presence of HSRs in chromosomes 11, 12, and 13 in each of the following three corals, *C.aspera*, *E.aspera*, and *P.contorta*. Interestingly, based on our recent studies on five stony coral species, including *F.pentagona*, HSRs are commonly found in the coral chromosomes of non-*Acropora* species. This indicates that the presence of an HSR is a cytogenetic characteristic of certain taxa of stony corals (Taguchi et al. 2013, 2014, 2016, 2017, 2020; Vacarizas et al. 2021).

The FISH marker for *histone H3* gene was observed at the centromeric region of chromosome 1. The core histone genes are highly conserved and repetitive, and their loci can thus be detected using FISH probes containing the sequence of a single array composed of tandem repeats (Huang et al. 2011). Previous molecular cytogenetic study on stony coral *Acroporapruinosa* has shown that the core histone gene locus which contains the H2a and H2b sequences were on the centromeric region of chromosome 8 (Vacarizas et al. 2021). These results imply that the chromosomal location of a highly conserved core histone varies across different family (Acroporidae and Merulinidae) of order Scleractinia (stony corals).

Information on 5S rDNA gene among stony corals is very limited (Taguchi et al. 2017; Taguchi et al. 2020; Vacarizas et al. 2021). We therefore developed the 5S rRNA gene primers reported not only in scleractinian corals, but also from other distant taxa such as hydra, starfish, and jellyfish (Hori et al. 1982; Walker and Doolittle 1983; Hendriks et al. 1987; Stover and Steele 2001) in which cytogenetic studies have identify specific FISH markers for 5S rRNA gene. The 5S rRNA gene primer from *Hydra* utilized by Stover and Steele (2001) were used to amplify suitable FISH probe sequences for this coral *F.pentagona*. Surprisingly, the sequence contain not only sequence of 5S rRNA gene but also the complete region of the U2-snRNA. The 5S rRNA gene and the U2-snRNA gene sequence were homologous to those of *C.aspera* (LC120341) and *A.pruinosa* (LC557013), respectively (Fig. 6). In this study, we showed that chromosomal location of this U2 snRNA-5S of *F.pentagona* was near the telomere region of chromosome 2. This is different from the chromosomal location of 5S-snRNA in *C.aspera* (Taguchi et al. 2017) and *A.pruinosa* (Vacarizas et al. 2021) chromosomes. Based on the alignments (red rectangle in Fig. 7), several insert nucleotide sequences were found only in the *U2 snRNA* gene of *F.pentagona*. This suggests that *U2 snRNA* and *5S rRNA* genes seemed to be fused, as cloning-sequence analysis and the sequence of the cloned insert is a part of the pseudogene.

Each of the FISH markers derived from the four different amplicons (FP-18S, FP-H3, FP-U2-5S, and FP-X9) were observed at a single site (locus), even in uncontracted prometaphase spreads with elongated chromosomes. As the specificity of the markers is very high due to the highly repetitive and conserved nature of these genes, these markers will be useful for karyotyping and identifying specific chromosomes containing similarity of sequences within closely related species.

## ﻿Conclusion

The series of cytogenetic studies on stony corals including the development of specific FISH markers may contribute to understand changes in chromosomal structures across taxa thereby gain insight into its evolutionary processes (Guo et al. 2018). Thus, these results might shed light on coral diversity and improve the classification of corals. However, it is important to develop more suitable FISH markers which can identify specific gene loci. In this study, we isolated FISH markers that provided distinct and bright hybridization signals on chromosomes that suitable for routine observation.

We highlighted the development of four chromosomal FISH markers for U2 snRNA-5S, 18S rRNA, histone H3, and uncharacterized FP-9X genes, which were found to be located on chromosomes 2, 10, 1, and 9, respectively. The loci of the gene histone H3 were mapped in the chromosomes of stony corals for the first time. The isolation of four FISH markers for *F.pentagona* will also promote gene mapping and understand genome structure and organization for this species. This cytogenetic information on stony coral along with morphological and molecular characteristics may contribute to understand its evolutionary processes and resolve taxonomic problems in stony coral taxonomy.

## ﻿Authors’ contribution

TT: designed and performed experiments, organized the figures, and drafted manuscript. RK, MI, and JV: performed experiments, organized the figures and the tables. TM: did sampling and identification of the specimens. RK and JV: corrected the manuscript. AT, SK, and TT: supervised the research and corrected the manuscript. All authors read, discussed, and approved the final version of the manuscript.

## References

[B1] Biedler JL Spengler BA (1976) Metaphase chromosome anomaly: association with drug resistance and cell-specific products. Science 16(191): 185–187. 10.1126/science.942798 942798

[B2] Fukami H Budd AF Paulay G Solé-Cava A Chen CA Iwao K Knowlton N (2004) Conventional taxonomy obscures deep divergence between Pacific and Atlantic corals. Nature 427: 832–835. 10.1038/nature02339 14985760

[B3] Gokhman VE Cioffi MB König C Pollmann M Gantert C Krogmann L Steidle JLM Kosyakova N Liehr T Al-Rikabi A (2019) Microdissection and whole chromosome painting confirm karyotype transformation in cryptic species of the *Lariophagusdistinguendus* (Förster, 1841) complex (Hymenoptera: Pteromalidae). PLoS ONE 14(11): e0225257. 10.1371/journal.pone.0225257 PMC6855445 31725808

[B4] Guo L Accorsi A He S Guerrero-Hernández C Sivagnanam S McKinney S Gibson M Alvarado AS (2018) An adaptable chromosome preparation methodology for use in invertebrate research organisms. BMC Biology 16: e25. 10.1186/s12915-018-0497-4 PMC5828064 29482548

[B5] Hendriks L De Baere R Vandenberghe A De Wachter R (1987) The nucleotide sequences of 5S ribosomal RNA of *Actiniaequine* and *Sepiaofficinalis*. Nucleic Acids Research 15: e2773. 10.1093/nar/15.6.2773 PMC340685 2882478

[B6] Hori H Ohama T Kumazaki T Osawa S (1982) Nucleotide sequences of 5S rRNAs from four jellyfishes. Nucleic Acids Research 10: 7405–7408. 10.1093/nar/10.22.7405 6130512 PMC327013

[B7] Huang D Licuanan WY Baird AH Fukami H (2011) Cleaning up the ‘Bigmessidae’: Molecular phylogeny of scleractinian corals from Faviidae, Merulinidae, Pectiniidae and Trachyphylliidae. BMC Evolutionary Biology 11: e37. 10.1186/1471-2148-11-37 PMC3042006 21299898

[B8] Kawaida H Shimizu H Fujisawa T Tachida H Kobayakawa Y (2010) Molecular phylogenetic study in genus *Hydra*. Gene 468: 30–40. 10.1016/j.gene.2010.08.002 20708072

[B9] Levan A Fredga K Sandberg AA (1964) Nomenclature for centromeric position on chromosomes. Hereditas 52: 201–220. 10.1111/j.1601-5223.1964.tb01953.x

[B10] Rowan R Powers DA (1992) Ribosomal RNA sequences and the diversity of symbiotic dinoflagellates (zooxanthellae). Proceedings of the National Academy of Sciences of the United States of America 89(8): 3639–3643. 10.1073/pnas.89.8.3639. 1565660 PMC48924

[B11] Seabright M (1973) Improvement of trypsin method for banding chromosomes. Lancet 2: 1249–1250. 10.1016/S0140-6736(73)90561-8 4122594

[B12] Stover NA Steele RE (2001) Trans-spliced leader addition to mRNAs in a cnidarian. Proceedings of the National Academy of Sciences of the United States of America 98: 5693–5698. 10.1073/pnas.101049998 11331766 PMC33275

[B13] Sullivan BA (2020) A sampling of methods to study chromosome and genome structure and function. Chromosome Research 28: 1–5. 10.1007/s10577-020-09629-y 32157563 PMC7185174

[B14] Super M (1991) Reviews in Medicine: Medical genetics. Postgraduate Medicine Journal 67: 613–631. 10.1136/pgmj.67.789.613 PMC2399073 1924046

[B15] Taguchi T Bellacosa A Zhou JY Gilbert DJ Lazo PA Copeland NG Jenkins NA Tsichlis PN Testa JR (1993) Chromosomal localization of the Ox-44 (CD53) leukocyte antigen gene in man and rodents. Cytogenetics and Cell Genetics 64: 217–221. 10.1159/000133580 8404042

[B16] Taguchi T Kubota S Mezaki T Sekida S Okuda K Nakachi S Shinbo S Iiguni Y Tominaga A (2013) Detection of characteristic heterochromatin distribution, highly amplified rRNA genes and presence of the human satellite III DNA motif in the scleractinian coral *Echinophylliaaspera* Ellis and Solander 1788. Chromosome Science 16: 33–38.

[B17] Taguchi T Mezaki T Iwase F Sekida S Kubota S Fukami H Okuda K Shinbo T Oshima S Iiguni Y Testa JR Tominaga A (2014) Molecular cytogenetic analysis of the scleractinian coral *Acroporasolitaryensis* Veron & Wallace 1984. Zoological Science 31: 89–94. 10.2108/zsj.31.89 24521318

[B18] Taguchi T Kubota S Mezaki T Tagami E Sekida S Nakachi S Okuda K Tominaga A (2016) Identification of homogeneously staining regions by G-banding and chromosome microdissection, and FISH marker selection using human Alu sequence primers in a scleractinian coral *Coelastreaaspera* Verrill, 1866 (Cnidaria). Comparative Cytogenetics 10: 61–75. 10.3897/CompCytogen.v10i1.5699 27186338 PMC4856926

[B19] Taguchi T Kubota S Tagami E Mezaki T Sekida S Okuda K Tominaga A (2017) Molecular Cytogenetic Analysis and Isolation of a 5S rRNA-Related Marker in the Scleractinian Coral *Platygyracontorta* Veron 1990 (Hexacorallia, Anthozoa, Cnidaria). Cytologia 82(2): 205–212. 10.1508/cytologia.82.205

[B20] Taguchi T Tagami T Mezaki T Vacarizas JM Canon KL Avila TN Bataan DAU Tominaga A Kubota S (2020) Karyotypic mosaicism and molecular cytogenetic markers in the scleractinian coral *Acroporapruinosa* Brook, 1982 (Hexacorallia, Anthozoa, Cnidaria). Coral Reefs 39: 1415–1425. 10.1007/s00338-020-01975-x

[B21] Takaoka E Sonobe H Akimaru K Sakamoto S Shuin T Daibata M Taguchi T Tominaga A (2012) Multiple sites of highly amplified DNA sequences detected by molecular cytogenetic analysis in HS-RMS-2, a new pleomorphic rhabdomyosarcoma cell line. American Journal of Cancer Research 2: 141–152. 22432055 PMC3304565

[B22] Testa JR (1990) Chromosome translocations in human cancer. Cell Growth Differentiation 1: 97–101. 2085464

[B23] Vacarizas J Taguchi T Mezaki T Okumura M Kawakami R Ito M Kubota S (2021) Cytogenetic markers using single sequence probes reveal chromosomal locations of tandemly repetitive genes in scleractinian coral *Acroporapruinosa*. Scientific Reports 11: e11326. 10.1038/s41598-021-90580-1 PMC8167085 34059722

[B24] Veron JEN (2000) Corals of the World. Vol 3. Townsville: Australian Institute of Marine Sciences, 1410 pp.

[B25] Walker WF Doolittle WF (1983) Nucleic Acids Research 11: 5159–5164. 10.1093/nar/11.15.5159 PMC326244 6136024

[B26] Wallace CC (1999) Staghorn Corals of the World. CSIRO Publishing, Collinwood, 421 pp. 10.1071/9780643101388

